# A dynamical measure of algorithmically infused visibility

**DOI:** 10.1098/rsos.242232

**Published:** 2025-11-26

**Authors:** Shaojing Sun, Zhiyuan Liu, David Waxman

**Affiliations:** ^1^ School of Journalism, Fudan University, Shanghai, People’s Republic of China; ^2^ Centre for Quantitative Biology, ISTBI, Fudan University, Shanghai, People’s Republic of China

**Keywords:** social media, communication, computer–human interaction

## Abstract

This work focuses on the nature of visibility in societies where the behaviours of humans and algorithms influence each other—termed algorithmically infused societies. We propose a quantitative measure of visibility, with implications and applications to an array of disciplines including communication studies, political science, marketing, technology design and social media analytics. The measure captures the basic attributes of the visibility of a given topic in algorithm-mediated communication settings associated, for example, with social media. These attributes are: (i) the amount of time a topic spends at different ranks and (ii) the different ranks the topic attains. In addition, the proposed measure incorporates a tunable parameter, termed the discrimination level, whose value determines the relative weights of the two attributes that contribute to visibility. The proposed measure is applied to the Hot Search List of the Chinese microblog Sina Weibo. Analysis of a large-scale, real-time dataset of topics of this list demonstrates that the proposed measure can explain a large share of the variability of the accumulated views of a topic.

## 1. Introduction

Human beings are entering an ‘algorithmically infused society’, namely a society that is increasingly being shaped by the combined behaviours of humans and algorithms/artificial intelligence (AI) [1]. Algorithms are not just employed in the selection and distribution of information, but are *dynamic actors* that have the effect of organizing and shaping behaviour at the level of the individual, and also at the level of society as a whole. As an illustration of this, Guess *et al*. [2] recently demonstrated that the feed-ranking algorithms of Facebook and Instagram had a strong influence on the experience of users of these two platforms, and a ‘reverse-chronologically ordered feed’ dramatically reduced the time users spent and decreased their engagement with content. It seems that the complex role that algorithms play in the dissemination of information requires a rethinking of communication processes that are often entangled with the ‘properties of algorithms, the relationality of algorithms and the temporality of the materialization of algorithms’ [3].

In various aspects of our lives, algorithms are becoming more pervasive and often affect what is seen, recognized and even remembered. The role that algorithms play in the mediation of information has profound implications for the dissemination of information, public discourse, individual experiences and societal dynamics. Phenomena that are of growing concern, such as filter bubbles, echo chambers, clickbait, marginalized visibility, to name a few, are all intimately connected with the action of algorithms. It seems fair to say that algorithms are exhibiting an increasing impact on what, to the general public, is visible (and what is invisible).

Although ranking is common across various societal sectors (e.g. academia, sports and marketing), an emerging trend is that algorithmic ranking has increasingly become a platform-mediated signal. For instance, social media platforms often use algorithms to prioritize content, rendering ranking a direct signal of algorithmic visibility. Unlike cumulative views (which are user-driven), algorithmic ranking reflects platform design choices (e.g. TikTok’s ‘velocity’ score, Google’s ‘freshness’). As such, there is a need to bridge the gap between rank data, platform dynamics and theories about measuring visibility [4].

In this work, we propose a dynamical metric of rank-based visibility that ‘adds value’ to the typical metric of view counts in the following aspects:

(1) View counts measure user attention, while our proposed measure may allow decoding of the way platforms gatekeep visibility and reveal algorithmic intent (platform prioritization). In other words, our visibility measure may expose whether visibility is ‘earned’ (user-driven) or ‘granted’ (algorithmically amplified). Understanding the importance of these aspects of visibility is crucial for auditing platform bias.
(2) As will become clear, our proposed measure of visibility distinguishes between ‘velocity’ (steep trajectories, denoting virality) and ‘endurance’ (stable trajectories, denoting community building) and captures temporal dynamics by integrating these two attributes.
(3) User views are platform-specific, whereas our proposed measure enables cross-platform comparison of visibility by normalizing rank decay.
(4) User views can be *gamed* (e.g. by bots, click farms) and hence can be contextually misleading, whereas our proposed measure has the potential to flag manipulation or misalignment between platform curation and user interest.
(5) Even for cases where visibility must be inferred from rank alone (i.e. actual view data are unavailable), our proposed measure may shed light on the complex relationships between content features, spatial positioning, temporal shifts and social–technical powers (e.g. political influence and platform manipulation). Furthermore, the measure of visibility that we propose in this work may enable researchers to reverse-engineer platform operations (e.g. addressing questions about whether a platform’s algorithm favours sudden bursting or sustained engagement in attracting user attention, and the way such a preference varies across topical domains).

## 2. Conceptualizing visibility in digital space

The visibility (seeing) of others might initially be described as involving ‘those who share the same spatial-temporal locale’ and is reciprocal in the sense that ‘we can see others who are within our field of vision, but they can also see us’ [5]. However, advances in communication technology have greatly reshaped our understanding of visibility, and new forms and properties of visibility have arisen. In the digital context, visibility is freed from spatial and temporal constraints of the here and now and involves scenarios such as ‘there and then’ or ‘there and now’. In other words, in a media-saturated society, visibility has become increasingly mediated by communication. Researchers in this area have proposed new concepts such as *mediated visibility* and *socially mediated visibility* [6] to describe the enriched and evolving nature of visibility. Treem & Leonardi [7] suggested that visibility is a kind of affordance, and from this perspective, visibility signifies the possibility of certain actions (e.g. the likelihood of a message to be viewed), and the higher the visibility, the higher the possibility for actions such as clicking or viewing to occur. Following this logic, it stands to reason that the more visible a trending topic is, in digital space (like a ranking list), the more likely individual users are to perceive such affordances, and the more they will tend to actualize such affordances by clicking or reading the topic.

Flyverbom *et al*. [8] proposed the concept of *visibility management* to explain information flow and control in digital space. Although those authors primarily defined the concept from an organizational and managerial perspective, the notion of visibility management relates to algorithm-sustained recommendation/ranking of, for example, the *Hot Search List* (HSL) of the Chinese microblog Sina Weibo. We argue that the phenomenon of the ranking of the HSL can also be considered visibility management, i.e. a dynamical social process that includes the interplay of social media organizations, technologies, individuals and governments. As such, the dynamic, relational and ambiguous workings of vision speak to the power of related stakeholders as well as the management of information flow.

The meaning of visibility has become even more complicated as algorithms begin to exercise an increased impact on the phenomenon. For example, Rieder *et al*. [9] looked into the operation and consequence of the YouTube search algorithm, from a social–technical perspective. Those authors argued that ranking algorithms are a complex process that unfolds over time, involving multiple actors such as advertisers, end users and politicians. As such, ranking algorithms are, in essence, ‘ranking cultures’ that are rendered as evolving processes that modulate visibility and network/coordinate different actors. Visibility, in digital media landscapes, is intricately linked to the attention and engagement of users. Digital platforms are designed to capture and hold users’ attention, and visibility often hinges on the ability to stand out and resonate with an audience in this highly competitive environment [10]. However, in an algorithm-mediated communication setting, visibility is much harder to define and quantify. The reason is that the algorithm-sustained digital space is not static, but fluid and continually changing. The complex mutual influences of algorithms and users make the space intrinsically dynamical. For example, the HSL on Weibo is an ontogenetic space, since it entails both being and becoming, with the listing and ranking of ‘hot’ topics continually changing. As such, measurement of digital visibility needs to take into account the fluid, evolving/dynamical nature of the space.

Past research on visibility in digital space suggests that visibility is (i) relational and (ii) temporal. It is relational because a visible topic or event is closely connected to other content, users or events, as explicated by the actor network theory of Latour. When it comes to the HSL, there always seems to be a tension between the ‘promise of visibility’ and the ‘threat of invisibility’: a new topic may enter the list of highly ranked topics, and an existing one on the list may exit (effectively be pushed out). It is temporal because visible content does not remain in the spotlight, and hence remains visible all of the time. Rather, due to the algorithm’s moderation and a platform’s selection, a topic’s status, in the space of topics, fluctuates over time. In developing a measure of visibility, the relational and temporal aspects should be explicitly factored in. As Wagner *et al*. [1] argued, measurement of algorithmic-infused societies needs to improve the match between theoretical constructs and operational measures.

## 3. Measuring visibility in algorithmically infused societies

In recent years, social scientists have devoted a significant effort to studying the linkage between visibility and algorithm-sustained media. For example, in this context, Bucher [11] critically examined the newsfeed and the EdgeRank algorithm of Facebook in terms of

— *affinity*: the interaction between the content viewer and the creator;
— *weight*: an interaction with particular content is assigned a weight by the algorithm, depending on how important the interaction is judged to be;
— *time decay*: the recency of an interaction.

Despite mounting research on visibility in digital settings, there is a lack of coherent theorizing about visibility. Indeed, to date, there has been little research on how to empirically measure visibility. As a result, most studies rely on platform-provided indices (e.g. the number of likes) to assess visibility. Recently, Wagner *et al*. [1] explicated the value of developing valid measures to assess algorithmically infused societies. According to those authors, opacity, dynamicism, interconnectedness and heterogeneity often lead to ready-made indices being inadequate to capture algorithm-sustained communication processes. Wagner *et al.* contended that researchers should integrate theory-driven and data-driven approaches in order to develop high-quality and transparent measurements of algorithmically infused societies. To echo their call, we believe the development of a *quantitative measure* of visibility, in an algorithm-mediated communication setting, will be of great value.

There have been previous approaches to measuring changes in ranking (see the work of Rieder *et al*. [9] and that of Webber *et al*. [12]). Such approaches (e.g. a rank-biased distance metric) typically give more weight to changes at the top of the list than changes further down. However, to the best of our knowledge, prior metrics have not been tailored to a digital space, like trending topics on social media. Furthermore, sophisticated mathematical approaches often render those metrics formidable, if not inaccessible, to social science researchers. It is our goal in this work to present an accessible, readily computable, quantitative measure of visibility in an algorithmic ranking space, which captures changes of rank over time. In other words, visibility in the digital space is characterized by multiple changes over time, and a sound measure, in this case, needs to simultaneously reflect changes of rank. Moreover, following the call of Rieder *et al.* for a strategy of descriptive assemblage—which is focused on analysing ranking outcomes rather than seeking causality of algorithms—we create our measure by connecting two key attributes, which are the relative ranking position of a topic and the amount of time it spends trending at a given rank. The present study is, to the best of our knowledge, the first attempt to develop a theory-based measure of visibility that can readily employ data in the HSL space. Such an academic endeavour may advance theoretical innovations in conceptualizing visibility in a dynamic, systematic and complex digital context.

The data used in this work came from the Weibo platform (one of the largest Chinese social media platforms). The HSL of Weibo presents a list of popular topics that are updated on a minute-by-minute basis. In electronic supplementary material, figure S1, we give an annotated screenshot of the HSL space. The top 50 topics are numbered and presented in a top-down order.

The Weibo homepage explains that ranking is derived via an algorithmic index, named *hotness*—which is a calculated quantity based on a topic’s recency, viewership and significance. However, the precise way in which the index is computed, and the ranking created, is *opaque*. Generally, we deem a lack of transparency a common issue for such algorithmic spaces, as Rieder *et al*. [9] discussed, in terms of a descriptive-assemblage approach, to understand ranking culture.

For our study, we first needed to acquire data. We achieved this by creating a Web crawler that captured the topics on the HSL, every time point (every minute), on Weibo, the Chinese media platform. We obtained 5 666 450 data entries that covered 27 012 different topics for the period 17 December 2022 to 8 March 2023. We note that all data collected were not tied to information that allows identification of any individual, and all data were publicly available.

In the quantitative approach that we present next, when we describe a topic as *trending*, it means that at the time in question, the topic has a ranking in the ‘top 50’ (i.e. its rank has one of the values 1, 2,…, 50, with 1 the highest rank). This reflects the data that are publicly available on the platform (Weibo).

## 4. Proposing a quantitative measure of visibility

We now provide a description of the measure of visibility that we propose in this work. The fundamental objects we deal with are *rank trajectories* or simply *trajectories* of different topics. We write a trajectory of a particular topic as

**Table:** 

time	rank
*t*_*1*_	*R*_1_
*t*_2_	*R*_2_
*t*_3_	*R*_3_
⋮	⋮
*t_f_*	*R_f_*

In this table, *t*_1_ denotes the time (measured in minutes of the day) that the rank of a given topic *first* appears in the top 50 ranks (i.e. it lies in ranks 1, 2,…, 50). The rank of the topic that is recorded at the time *t*_1_ is denoted by *R*_1_. When the rank of the topic is next in the top 50 ranks, the time is denoted by *t*_2_ and the corresponding rank that is recorded is *R*_2_. Continuing in this way, the *final* time the topic lies in the top 50 ranks is denoted by *t_f_* and the corresponding rank that is recorded is *R*_*f*_.

In this way, the trajectory of any topic consists of:

(i) a list of times where the topic is in the top 50 ranks and
(ii) a list of the ranks that the topic attains at these times.

Note that times and the associated ranks are only recorded for ranks in the top 50. Thus, in the above table, the times may not be at every minute, and adjacent times in the list may not differ by unity. For example, in the above trajectory, we could have the times *t*_1_ = 7, *t*_2_ = 8 and *t*_3_ = 10. This means:

(i) the trajectory *started* (first entered the top 50 ranks) at time 7 min and its rank was recorded,
(ii) the trajectory was in the top 50 ranks at time 8 min and its rank was recorded,
(iii) at time 9 min the rank of the trajectory was *outside* the top 50 and its rank was *not* recorded,
(iv) at time 10 min the trajectory was again in the top 50 ranks and its rank was recorded.

## 5. Fundamental properties of a measure of visibility

Here, we list what we believe are the fundamental properties that *any* measure of visibility should have. We then present a quantitative measure of visibility that has these properties.

**Table:** 

trajectory 1		trajectory 2		trajectory 3		trajectory 4	
*time*	*rank*	*time*	*rank*	*time*	*rank*	*time*	*rank*
1	40	10	40	27	40	27	40
2	30	11	40	28	30	28	30
3	50	12	30	29	30	29	30
		13	30	30	50	30	50
		14	50	31	40	31	40
		15	50	32	50	32	20

(1) The visibility of a topic should be proportional to the time a topic is trending, *all other things being equal*. By this, we mean given the above four *complete trajectories*,^1^ a reasonable measure of visibility will accord trajectories 2 and 3 precisely *twice* the visibility of trajectory 1, because irrespective of the order of appearance of the ranks, and the times that the topic is trending, trajectories 2 and 3 achieve all of the ranks that trajectory 1 achieves, but trajectories 2 and 3 spend *twice* as much time at these ranks as trajectory 1.
(2) Times of high rank should contribute more than the corresponding times of low rank. For example, if there are two topics whose trajectories have the same length, and they have ranks that agree at all except a single time, then the trajectory with the higher rank at this time will generally have a higher visibility. For the trajectories given above, trajectory 4 will generally have a higher rank than trajectory 3 because although trajectories 3 and 4 have the same length, trajectory 4 has one time point (at minute 32) where its rank exceeds that of trajectory 3.

To proceed, we introduce a quantity D, that we term the *discrimination level*, which intuitively captures the way we can distinguish between different ranks, as we shall shortly explain. Then, in accordance with the two assumptions given above, we propose that the *visibility* of a topic, at a discrimination level of D, written V(D), is given by


(5.1) $$V(D)=\sum_{i=1}^{f}\frac{1}{R_{i}^{D}},$$

where the sum is over all time-points where the rank of a topic has been recorded (i.e. lying in the top 50 ranks). The discrimination level, D, takes values that are non-negative (D≥0). We shall present results when D is restricted to a finite range, for example


(5.2) 0≤D≤3.

When the discrimination level has the value zero (D=0), we say there is *no discrimination*, in the sense that the corresponding visibility, V(0), measures the total time the topic is trending (i.e. V(0) is simply the *number of minutes* that the topic spends in ranks 1–50, irrespective of the value of the rank). For the trajectories 1, 2, 3 and 4, given above, the value of V(0) is 3, 6, 6 and 6, respectively, because these are simply the number of times where the topic is trending.

When, for example, D=3, there is a *very high level of discrimination*, in the sense that every minute that a topic spends at rank 1 contributes *eight* times as much as every minute where the topic is at rank 2, and 27 times as much as every minute where the topic is at rank 3.

Psychology studies have shown that humans shift their attention from one location to another when viewing a complex image, given the limited capacity of the human visual system in simultaneously processing multiple cues [13]. That said, a person is more likely to allocate more attention to a salient target in the scene and much less attention to nonsalient targets. According to past research on saliency ranking in computer vision, a user’s ranking of the salience of objects in a scene does not follow a linearly decreasing process. It is plausible that the top object is accorded a much higher weight than those further down the list [14]. In the language of visibility, given in equation (5.1), this might correspond to a somewhat appreciable value of D.

However, even a discrimination level of D=1 is somewhat discerning in the sense that for the trajectories 1, 2, 3 and 4 given above, the values of V(1) are, to four decimal places, 0.0783, 0.1567, 0.1567 and 0.1867, respectively. Thus, at a discrimination level of D=1, trajectory 4 has a visibility that exceeds that of trajectories 2 and 3 by more than 19%, due to the occurrence of a single time where trajectory 4 has a higher rank.

Discrimination levels that are higher than those we have considered (e.g. D>3) are possible, and these can expose other aspects of a topic; for example, if we allow D to become arbitrarily large (D→∞), then the resulting value of the visibility just counts the amount of time the topic spends at rank 1.

We note that there is a simple geometric interpretation of the visibility, V(D). For a given value of the discrimination level, D, we can transform a trajectory, with rank and hence ‘height’ R, at a given time, to a new trajectory, with transformed height $1∕R^{D}$, which represents a measure of the *importance* of the rank attained at the given time, since higher ranks are more important than lower ones. Taking the importance of a topic as *zero* for ranks outside the top 50, the *area* under this transformed ‘importance’ trajectory, for the given discrimination level, is precisely our visibility measure. Thus, our visibility measure is the *accumulated importance of a topic over the time it is trending*.

**Figure 1.**
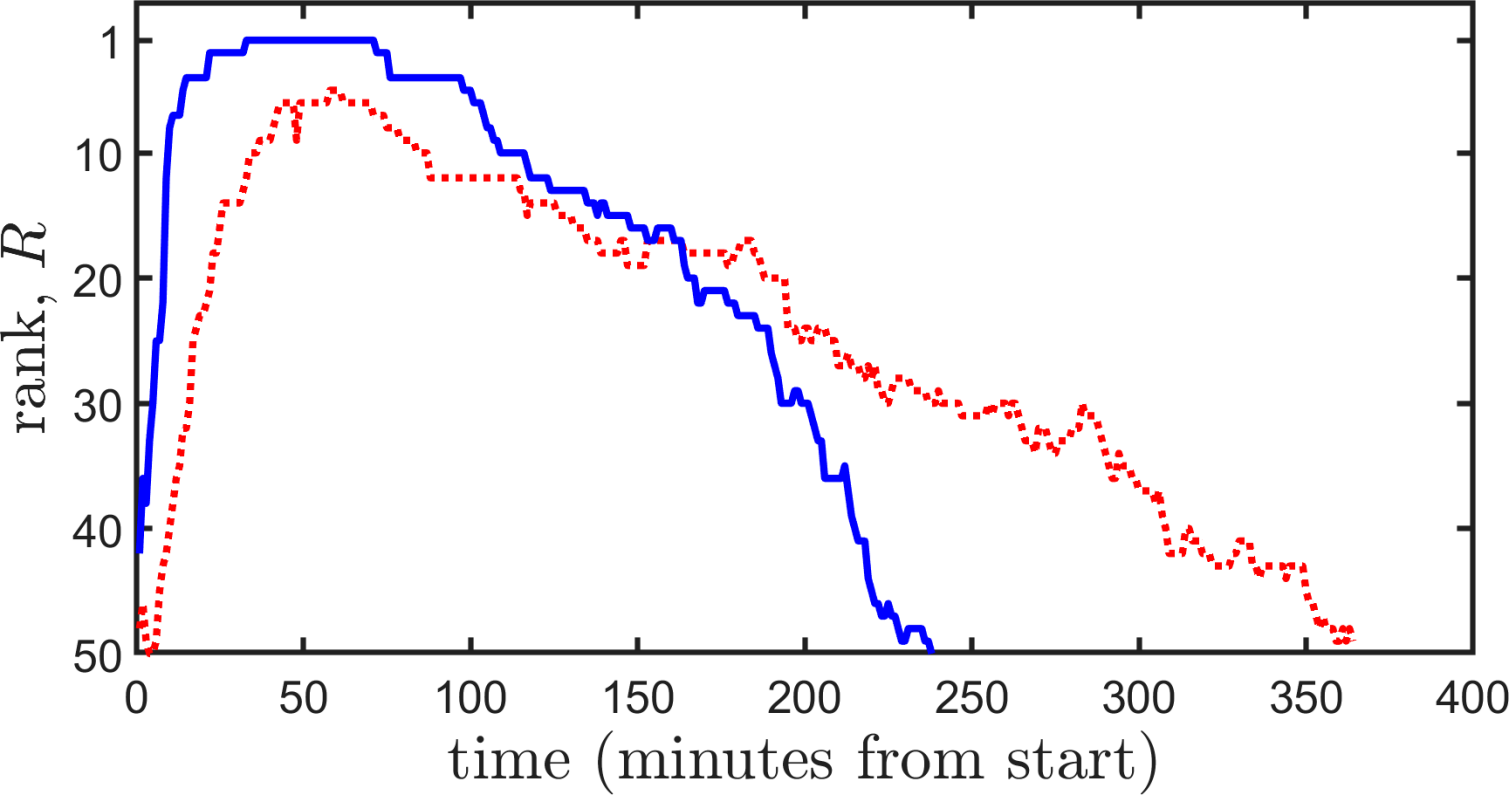
Trending trajectories. We plot the trajectories (rank, *R*, versus time trending) of two different topics. One topic attains a higher maximum rank than the other, while the other topic trends for a longer period of time. To aid visualization, we subtracted, for each trajectory, the initial time from the actual times the rank was observed. This allows us to plot both trajectories from a common origin (0) and has no effect on the visibility of the two trajectories.

**Figure 2.**
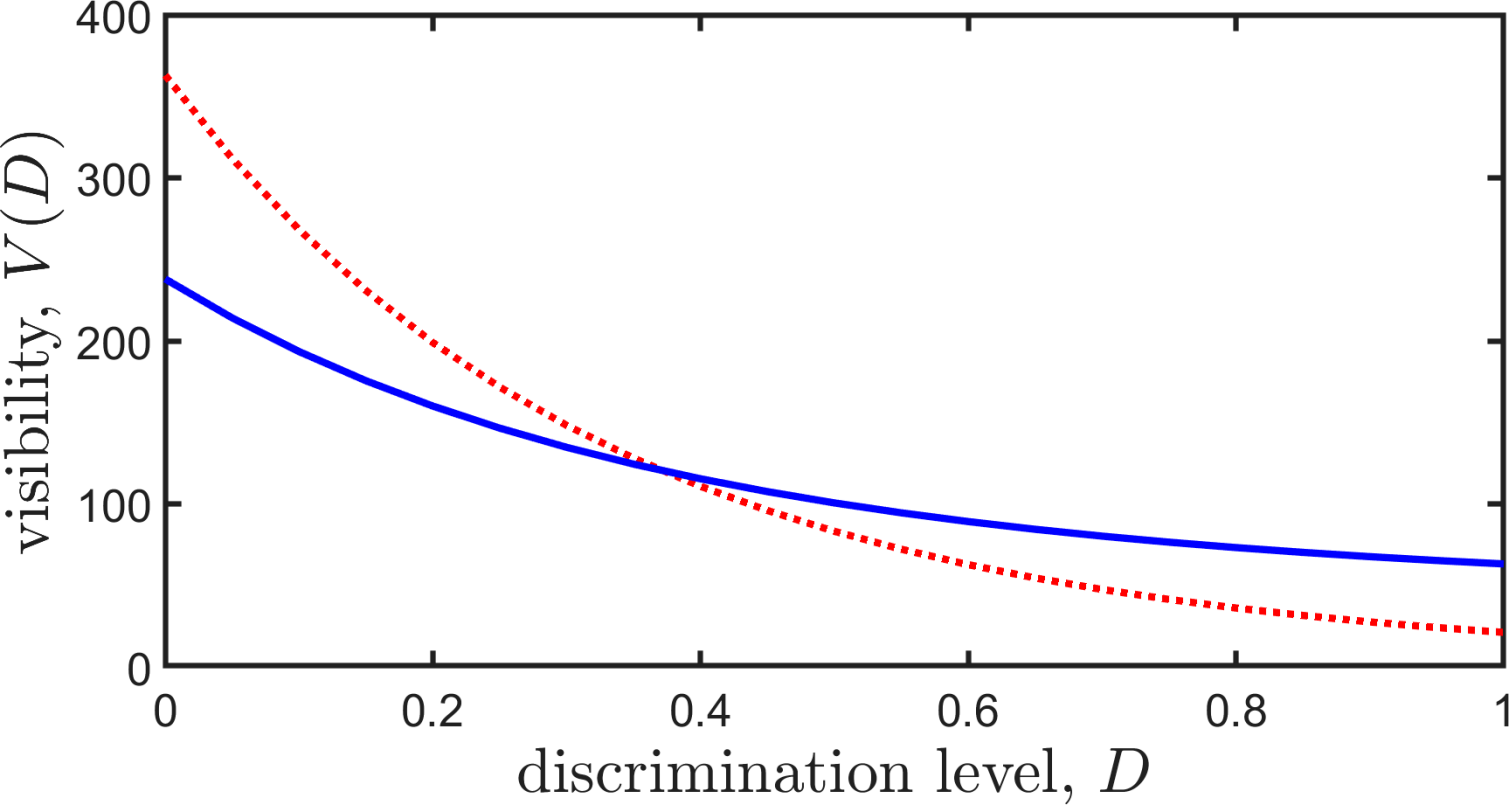
Visibility versus discrimination level. We plot visibility, *V(D)*, versus discrimination level, *D*, for two topics whose trajectories are given in figure 1. The two topics spend different amounts of time trending: the values of their visibilities at *D=0* indicate these different values (one topic trends for 363 min, the other for 238 min). For low discrimination levels, the topic with the longer trending time has a higher visibility. However, for sufficiently large *D (D≳0.37)*, the other topic has a visibility that exceeds that of the first.

In figure 1, we plot the trajectories of two different topics, and in figure 2, we plot their visibilities, as a function of discrimination level, D.

The two trajectories in figure 1 achieve maximum ranks that differ and have total trending times which differ. It is evident from figure 2 that at smaller discrimination levels, D, the longer-lived (longer trending) trajectory has higher visibility. However, for sufficiently large D (D≳0.37), the shorter-lived, but higher rank achieving trajectory has a visibility that exceeds that of the longer-lived trajectory. This indicates that at an appropriately high discrimination level, time spent at higher ranks more than counters a longer trending time. According to different criteria of visibility, i.e. for different choices of D, either of the two topics used for figures 1 and 2 may have a higher level of visibility.

## 6. Linking the visibility measure to outcomes

Algorithm-generated ranking of online content has proven to be linked to attention allocation and various behavioural outcomes. For instance, Pan [15] found that online search engine ranking of tourist destinations has a significant impact on *click-through rates* (CTRs). Specifically, the top-ranked results collected high CTRs, but the rates decreased precipitously with decreasing rank. In reference to previous literature, we reason that visibility, factoring in both ranking and temporality, should be linked to clicks, views and other behavioural outcomes, even though those outcomes may not solely depend on the impact of visibility. For instance, Lu & Pan [16] recently found that the Chinese government uses clickbait to compete for visibility of propaganda contents on *WeChat* (a major Chinese social media platform that is broadly equivalent to Facebook). Those authors found that placing hyperbolic words and exclamation marks in the titles had large and statistically significant effects on the number of views of the corresponding articles.

**Figure 3.**
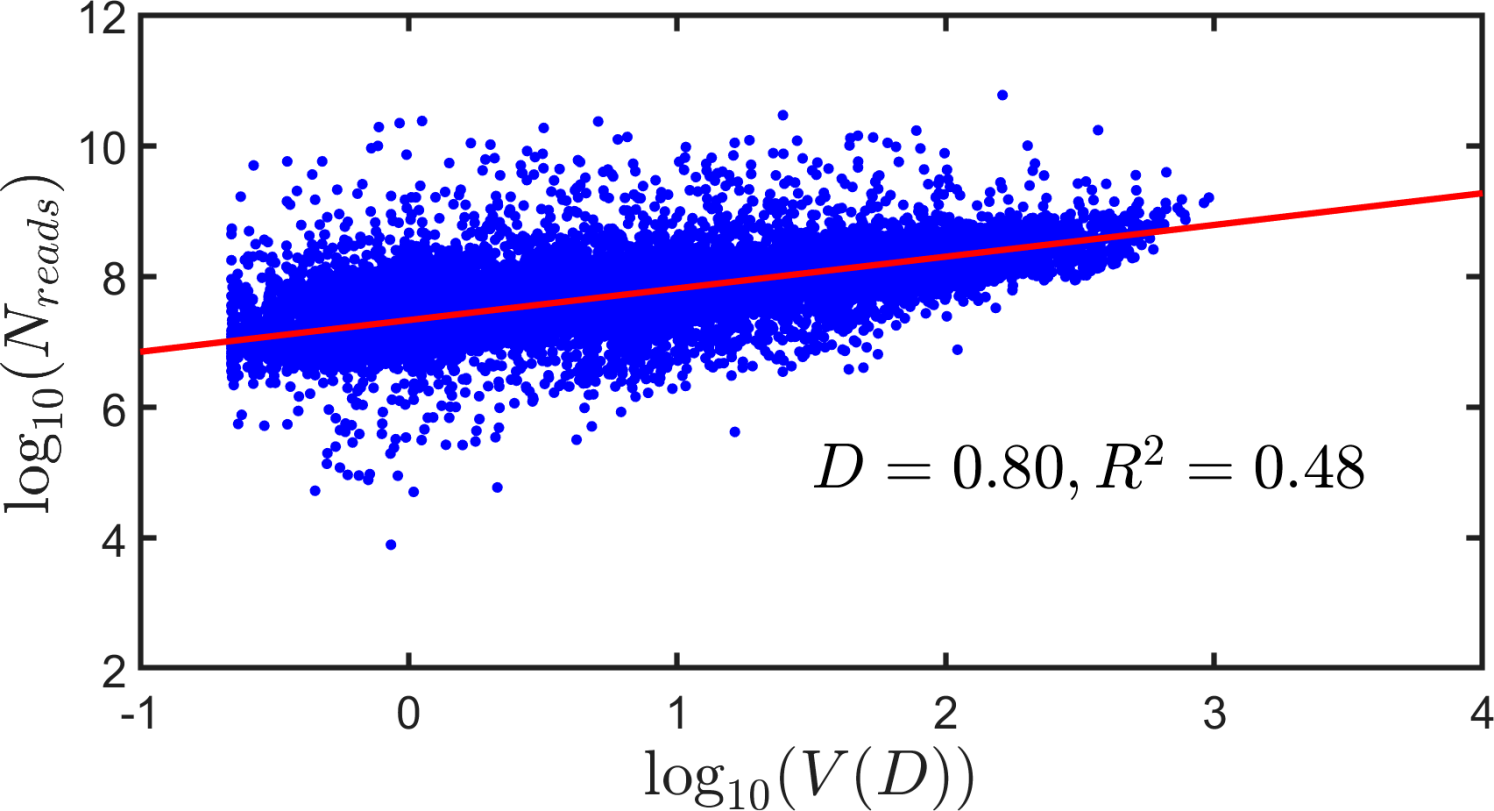
Scatter plot showing *$N_{reads}$* versus visibility. The figure contains a scatter plot of *$\log_{10}(N_{reads})$* against *$\log_{10}(V(D))$* for points associated with 23 993 different topics. The figure also contains the best straight line through the data (red). A value of the discrimination level of *D* = 0.80 was arbitrarily adopted, and the best straight line through the data yielded a value of *$R^{2}$* (coefficient of determination) of 0.80.

It is of interest to consider whether our proposed measure of visibility links to, explains or predicts certain outcomes. To this end, consider a particular topic and a given discrimination level, D. The corresponding visibility, V(D), can be calculated from knowledge of the set of ranks the topic attains over time. There are various criteria or indices associated with the topic, of which an important one is the *total number of reads of the topic*. With


(6.1) $$N_{reads}=\text{total number of reads}$$

we give, in figure 3, a scatter plot of the ‘number of reads’, $N_{reads}$, against visibility, V(D), using logarithmically scaled axes. Data on more than 23 000 topics were used in the plot. Also plotted in the figure is the best straight line through the data (red).

**Figure 4.**
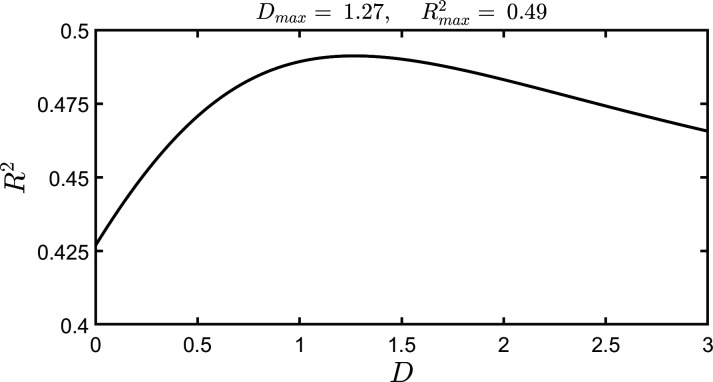
Coefficient of determination versus discrimination level. We determined the best straight line of *$\log_{10}(N_{reads})$* against *$\log_{10}(V(D))$*, for 23 993 different topics, for a set of different values of the discrimination level, *D*. The plot contains the *$R^{2}$* values of these lines against the corresponding values of *D*. We determined the value of *D* that maximizes *$R^{2}$*, and we write these quantities as *$D_{max}$* and *$R_{max}^{2}$*, respectively.

As we have already stated, the level of discrimination, D, does not have a predetermined value, but may be chosen according to the particular application of visibility that is envisaged. We can, however, look for the value of D that results in visibility *best explaining* the platform-supplied index—here the total number of reads of a topic. To search for this value of D, we first chose a finely spaced set of D values,^2^ and for each value of D in the set, we fitted a straight line through the logarithmically transformed data, as we did in figure 3—in that case, just for *D* = 0.80. We determined the value of the ‘coefficient of variation’, $R^{2}$, for each such fitted line, and hence for each such D value in the set. This yields figure 4, where it is observed that $R^{2}$ achieves a maximum value, written $R_{\text{max}}^{2}$, at an intermediate value of D. The maximum is well defined. We write the value of D corresponding to $R_{max}^{2}$ as $D_{max}$. Then, for any topic, $\log_{10}(V(D_{max}))$ is the logarithm of the visibility that best explains the variation in the logarithm of the total number of reads of the topic.

It is possible that not all minutes contribute equally to visibility, and time-based weighting should account for fluctuations in viewer counts across the day [17]. To investigate this, we *zero-weighted* the low-activity time interval, from 12 midnight to 07.00. To a very good accuracy, our original findings are robust, in the sense that the modified dataset, with the zero-weighted time interval, led to only minor changes from the values reported for the complete (unweighted) dataset, e.g. the change in $D_{max}$ between the two datasets was approximately 3%, and to two decimal places, $R_{max}^{2}$ was unchanged at 0.49.

Up to now, we have treated all topics as having an equivalent status. In point of fact, topics may be grouped into *categories* because the trending topic space on Weibo features a variety of topics. The Weibo platform provides a classification of the trending topics presented in the ranking list. Specifically, the platform classified all the topics into 26 broad categories (this is the official classification presented in metadata format by the Weibo platform) including, but not limited to, domestic news, fashion, music, animation and sports. It should be noted that the distinction between certain categories may not be clear-cut, especially as the platform has not provided a detailed description of the categorization procedure. Also, notably, the distribution of topics across the categories is markedly imbalanced, with some categories (e.g. music) featuring several thousand topics whereas others (e.g. film and TV series) feature only a dozen. To further illustrate the validity of our measure, we have repeated the above analysis by comparing the results across topical categories.

Our results show that the optimal parameter values vary across these categories/subcultures and hence indicate that ranking results are not solely determined by the algorithm adopted; rather, the outcome is contingent on the interplay between the algorithm and the culture it is embedded within. As Rieder *et al.* argued, the modulation of visibility on social media is more about ‘ranking cultures’ than ‘ranking algorithms’ [9].

The results are summarized in table 1, with a complete list of all 26 categories, along with some of their properties, given in electronic supplementary material, table S1. The results show that for HSL topics about sports and domestic news, the quantification of visibility, by our measure, can explain more than 65% of the variability of the accumulated viewership. By contrast, for HSL topics about social news, visibility can explain approximately 15% of the variability of the total readership. Due to the complex nature of the algorithmic space, we cannot jump to the conclusion that there is a cause-and-effect relationship between visibility and accumulated viewership. However, our results do show a relatively strong and statistically significant positive correlation between visibility of a topic and its total viewership. This provides some evidence that visibility is a kind of affordance for certain actions, such as clicking and reading in this case.

**Table 1.** Properties of major categories. Proportion of variation explained and maximum discrimination level for *major* topical categories (those containing more than 1000 topics).

category ID	$R_{\max}^{2}$	$D_{\max}$	no. of topics
humour	0.36	0.90	1549
sports	0.66	1.05	1209
domestic news	0.68	1.05	6108
music	0.53	1.20	4446
artists	0.35	1.65	2330
animation	0.52	1.80	1759
social news	0.15	1.80	1197

We draw attention to the fact that different categories of topics have different values of the maximum discrimination level, $D_{max}$. Some topics, such as those concerned with animation or social news, have values of $D_{max}$ that are as high as 1.80. By contrast, humour-related topics have a $D_{max}$ of around 0.90. These findings suggest that for topics such as animation, predicting the accumulated viewership is more connected with rank rather than the time spent at high ranks. By comparison, for topics such as humour, differences in rank are not as influential as the time spent in the top 50. We comment more on this in §7.

## 7. Discussion

In this study, we have proposed a quantitative measure to gauge visibility in the context of social media trending topics. The creation of the measure is rooted in theorizing visibility in the digital and especially algorithm-infused context. Our measure aligns with the relational, temporal and dynamic nature of visibility in the digital context. By taking into consideration the relative position of each topic’s rankings across time, we have proposed a measure with a discrimination parameter whose value is not set, but instead varies by research scenarios and needs. Such a manoeuvre is premised on the assumption that the visibility of an issue or entity is fluid and mutable in the digital space, because it is constituted by the social, technological, discursive and material processes [18].

In recent years, researchers have investigated the relationship between visibility and other cognitive/affective/behavioural outcomes from diverse angles. For example, Henderson *et al*. [19] departed from visual saliency theory and cognitive guidance theory to investigate how meaning and image saliency influence a person’s attention allocation in viewing real-world scenes. Those authors found that despite there being a significant impact of an image’s salience, cognitive relevance plays the dominant functional role in guiding and shaping attention allocation. If we view the HSL as a visual space, it is reasonable to argue that both the visual layout and cognitive relevance of those topics might affect the attention allocation and duration. In this study, we did not consider, in any detail, the semantic meaning of each topic. However, this may be a promising direction of future research.

Making comparisons in table 1, we note that sports news is characterized by a smaller value of $D_{max}$ and a larger value of $R_{max}^{2}$ than social news. This indicates a difference between the two types of news; sports news is more aligned with the time a topic is trending than its rank, while social news is largely the opposite. The large gap in our measure explaining accumulated viewership (i.e. the values of $R_{max}^{2}$)—65% for sports versus 15% for social news—can be viewed as a non-trivial feature of some topics that has been exposed by our study. It is clear that additional influences must be at play with social news, which means that although rank is emphasized for social news, there are other more important explanatory factors at play, with platform intervention as a possible example.

To better conceptualize visibility, it is necessary to tap into the operational nature and logic of digital settings such as trending topic recommendation and social media discussion. Duguay [20] demonstrated that the operation of Facebook trending topics is governed by social media logic entailing popularity, programmability, connectivity and datafication. For instance, this platform utilizes and displays volumes of user data (e.g. number of views) to create the impression that these trending topics are happening in real time. Duguay, furthermore, noted that the four aforementioned elements operate under the influence of an overarching logic (automation), rendering a perception that these trending topics are intervention-free and instantaneous.

Although our study has adopted a computational approach to craft a measure of visibility, future work should consider using mixed methodology to identify key factors driving or shaping visibility in digital communication. Ellison *et al*. [21], for example, found that on Facebook, user motivations and Facebook feed content are salient predictors of click behaviour. The authors distinguished four types of users including indiscriminate clicker (those with high frequency of clicking on social media content but without attentive and extended viewings), engaged clicker (those engaging in clicking and paying close attention to social media content), unengaged lurker (who rarely clicks or views social media content) and engaged lurker (who does not click much but does spend time viewing social media content).

As for the application of the proposed measure, we are agnostic to the values of D, but certain values may be adopted when particular applications are in mind (e.g. digital advertising). We can use the value of $D_{max}$ to expose differences of topics in different domains. In other words, we can use $D_{max}$ as a *partial classification* of different categories, which may function as an alternative way to tap into the differences in topics.

Some weaknesses of the current study should be noted. First, due to the constraints of data collection, we were only able to gather data about the aggregate readings of each topic. If researchers have a chance to collect data about the number of real-time readings across time, then that will provide a valuable opportunity to explore the correlations between our measure and users’ actual behavioural outcomes over time. Second, the categorization of the topics was derived from the platform-provided metadata. So, misclassification could be a potential problem. It would be of value to study how measured visibility explains user behaviours across topical domains based on a more rigorous classification scheme. The proposed visibility measure is based on two observable inputs—a topic’s rank and the amount of time it appears on the HSL, and it is validated against total view counts. However, it should be noted that on platforms like Weibo, a topic’s ranking, time on the list, and total view counts are all susceptible to manipulation. As Cui & Kertész [17,22] cautioned, ranks can be inflated or suppressed; the amount of time the topic remains on the HSL can be extended or truncated by platform to shape perception; and views can be artificially boosted by bots or click farms. As such, these hidden influences may undermine the neutrality of the inputs and, by extension, the validity of the measure. Put more bluntly, the metric may be quantifying a distorted version of visibility and validated against potentially manipulated outcomes. We acknowledge these potential limitations. However, those limitations do not deny the value of the present study, given the novelty of introducing the discrimination level parameter D in modelling algorithm-mediated visibility. We hope that future studies use alternative data sources (e.g. data from legacy media), research design (e.g. experiments), platforms (e.g. X, TikTok) to validate our measure.

In many real-world contexts (e.g. social media trending lists, academic citations, competitions), the difference in visibility between rank 1 and rank 2 is far greater than between rank 30 and rank 31. Our measure accounts for both the intensity (how high the ranks are) and persistence (how long they stay in the top list). In reality, visibility often decays nonlinearly over time. Even when view data are unavailable, our measure may have a high utility. For instance, in privacy-constrained social media contexts, our measure can provide a glimpse of the general landscape, even though the ranking could be affected, shaped or manipulated by multiple factors [17].

Theoretically, our measure formalizes the nonlinear relationship between ranking and visibility/power/impact, challenging the assumption that ranks contribute linearly or uniformly. Our measure treats rank trajectories as direct signals of algorithmic prioritization, operationalizing visibility through platform logic rather than user behaviour. Thus, the measure contributes to bridging the gap between ranking methodology and theories about attention dynamics. Specifically, researchers can harness our measure to test social and communication theories, e.g. do platforms with steep decay (large values of the discrimination level parameter D) amplify polarization? Does sustained mid-rank visibility (small values of the discrimination level parameter D) correlate with community trust?

Methodologically, our measure moves beyond static metrics and offers a tunable tool to test how different rank-weighting mechanisms affect conclusions. In this sense, our measure can function as a theoretically flexible proxy for visibility when view data are unobserved or unavailable. One practical example is about the impact of a scholarly publication, when conditional expected values such as E(views|rank) might assume a journal’s impact factor directly maps to the visibility of the publication. However, papers in the same journal may garner vastly different attention based on how long they maintain their top ranks (e.g. ranks in the ‘most read’ or ‘most cited’ lists). For example, in selecting and recommending articles to readers, instead of merely relying on an aggregate number of citations, journal editors can evaluate a pool of, say, 100 or 200 articles ranked by citations across a period of time, and then adopt a high D-parameter value to prioritize bursty citations, or use a low D value to favour enduring contributions. Our measure in this case can capture the nuanced temporal fluctuation in scholarly impact.

Developing quantitative measures of algorithmically infused societies is a challenging but invaluable task. Future research should continue to explore other concepts related to the digital setting and advance the development of sound measures that can better capture the nature of algorithm-mediated communication.

## Data Availability

All data relevant to this work have been submitted to Dryad [23]. Supplementary material is available online [24].

## Footnotes

^1^
A complete trajectory ranges from its initial time, *t*_1_, to its final time, *t*_*f*_.

^2^
The set of *D* values we adopted was 0.00, 0.01, 0.02,…, 3.00, since we aimed to provide results for the ‘best explaining *D*’ to two decimal places. Of course, the finer the spacing of *D* values, the longer the computations take.
